# Emergency physicians’ perceptions and optimization strategies for diagnostic imaging overuse in Somalia

**DOI:** 10.1186/s12873-026-01556-1

**Published:** 2026-03-24

**Authors:** Mohamed Mukhtar Mohamed, Fatima Mohamud Ahmed, Ibrahim Ağaoğlu, Sowdo Nur Iyow, Ibrahim Hussain Ali, Ömer Metin, Abdullahi Ahmed Ahmed

**Affiliations:** 1 Department of Emergency Medicine, Mogadishu Somali-Turkey Training and Research Hospital, Mogadishu, Somalia; 2 Faculty of Medicine and Health Sciences, Jamhuriya University of Science and Technology, Mogadishu, Somalia; 3 Faculty of Medicine and Surgery, Jazeera University, Mogadishu, Somalia; 4 Republic of Türkiye, Ministry of Health – Hittite University Çorum Training and Research Hospital, Çorum, Türkiye; 5 Republic of Türkiye, Ministry of Health – Ordu Ünye State Hospital, Ordu, Türkiye

**Keywords:** Diagnostic imaging, Overuse, Emergency medicine, Somalia, Defensive medicine

## Abstract

**Background:**

Overuse of diagnostic imaging in emergency medicine poses significant challenges, particularly in emergency care settings, where imaging practices are often influenced by diagnostic uncertainty and systemic factors. This study explored emergency physicians’ perceptions of unnecessary imaging, the key drivers of overuse, and potential strategies to optimize imaging.

**Methods:**

A prospective cross-sectional survey was conducted over a one-month period among emergency physicians working in the Emergency Department (ED) of the Mogadishu Somalia–Turkey Recep Tayyip Erdoğan Training and Research Hospital. A total population sampling approach was used; of the 25 physicians in the department, three were excluded after participating in the pilot phase, leaving 22 eligible participants. Data were collected using a structured, self-administered questionnaire consisting of 26 items across four domains: demographics, perceptions of imaging overuse, drivers of overuse, and optimization strategies. Most attitudinal items used Likert-type scales, and the survey included a single open-ended question asking physicians to identify the most important change their ED could implement to reduce unnecessary imaging. Data were analyzed descriptively using the Statistical Package for the Social Sciences (SPSS) version 24.

**Results:**

Twenty-one emergency physicians participated (81% male, 90.5% aged 20–35 years). All participants worked in a single tertiary teaching hospital. Over one-third (38.1%) reported ordering unnecessary tests daily. Main contributing factors included fear of missed diagnoses (66.7%), consultant pressure (57.1%), malpractice concerns (42.9%), and departmental culture (52.4%). Respondents identified physician education, malpractice reform, and shared decision-making as key strategies to mitigate overuse. Approximately half (47.6%) of the participants self-reported consistently using evidence-based decision rules.

**Conclusion:**

Overuse of imaging in Somali EDs is common and is primarily driven by defensive practices and systemic barriers rather than financial incentives. Physicians expressed strong interest in structured guidelines, educational initiatives, and audit-and-feedback systems to promote rational use of imaging.

## Introduction

Overuse in healthcare refers to the provision of diagnostic or therapeutic interventions in situations where there is little or no meaningful clinical benefit [1]. In emergency medicine, the excessive use of diagnostic imaging represents a major component of this problem [2]. Low-value imaging not only increases healthcare expenditures but also exposes patients to potential harm. Unnecessary tests can lead to incidental findings, overdiagnosis, and cascades of additional investigations or invasive procedures that offer limited clinical value [3–7]. These downstream effects may increase financial burden, prolong Emergency Department (ED) stays, and generate anxiety for patients and their families.

In modern EDs, the rapid expansion and accessibility of advanced imaging technologies have significantly transformed diagnostic workflows. Modalities such as computed tomography (CT) are increasingly used as frontline tools for patient evaluation and disposition decisions [8]. However, imaging rates vary widely among physicians and institutions for similar clinical presentations, indicating that utilization is influenced not only by patient-related factors but also by local practice patterns, resource availability, and individual decision-making styles [9, 10]. This variability underscores the importance of understanding how emergency physicians make imaging decisions in routine clinical practice and identifying opportunities to promote more appropriate, evidence-based use.

Beyond system-level and financial consequences, certain imaging modalities pose direct biological risks. Techniques that use ionizing radiation, such as X-rays and computed tomography (CT), have been associated with a small but measurable increase in the lifetime risk of cancer, particularly with repeated or unnecessary exposure [3–7]. In contrast, non-ionizing modalities such as ultrasound and magnetic resonance imaging (MRI) do not expose patients to ionizing radiation. However, MRI examinations are generally more costly, require longer acquisition times, and are often less accessible in many low-resource settings, which may limit their routine use in emergency care. The widespread and sometimes indiscriminate use of imaging, therefore, raises concerns about patient safety and resource allocation. Globally, low-value care, including unnecessary diagnostic testing, accounts for substantial annual expenditures without clear improvements in patient outcomes [11–13].

This global trend is particularly concerning in low- and middle-income countries (LMICs), where scarce resources worsen the impact of excessive testing. In these settings, the problem of unnecessary imaging is steadily expanding and is influenced by regulatory gaps, underenforced clinical protocols, and insufficient awareness of imaging guidelines [14]. Studies have shown that the rapid expansion of imaging technology, particularly in the private sector, has led to both underuse among disadvantaged populations and overuse among urban, insured, or wealthier groups, thereby further straining healthcare budgets [15]. Evidence gathered from low- and middle-income countries suggests that frequent imaging wastes limited funds and increases radiation exposure without providing added clinical benefit [16]. Research across African countries reveals high levels of unnecessary imaging referrals, approximately one in three cases, with guideline-based interventions in countries such as Uganda demonstrating significant reductions in misuse [17]. Similarly, utilization studies from South Africa have shown a steady rise in imaging volumes over the past decade, suggesting a growing risk of inefficiency and overuse in public-sector facilities [18].

Somalia is experiencing a growing concern about the excessive use of diagnostic imaging, particularly in the ED, where clinicians operate under time pressure and have limited access to consultations. The country’s healthcare system, which is still being rebuilt after decades of conflict, faces severe shortages of imaging resources, a lack of standardized imaging guidelines, and an uneven distribution of qualified personnel across institutions [19, 20]. These gaps create conditions in which advanced imaging may be overused for reassurance or to address diagnostic uncertainty, even when clinical evaluation might suffice. Despite widespread recognition of overuse as a healthcare problem, little is known about how emergency physicians in Somalia perceive or manage it. To date, no prior research has systematically examined this gap. This study aimed to assess emergency physicians’ perceptions of unnecessary diagnostic imaging, identify key drivers and barriers influencing overuse, evaluate the use of evidence-based decision tools, and explore strategies to optimize imaging practices in Somalia.

## Methods

### Study design

A prospective cross-sectional survey was conducted to capture emergency physicians’ perceptions of diagnostic imaging overuse, its contributing factors, and potential optimization strategies. A cross-sectional survey design was used because it is well-suited to exploring physicians’ attitudes, perceptions, and clinical practice patterns in a structured, systematic manner [21].

### Study setting

The study took place in the ED of the Mogadishu, Somalia–Turkey Recep Tayyip Erdoğan Training and Research Hospital, a tertiary-level teaching and referral facility that serves as the leading national referral center. The ED operates 24 h per day and manages a broad spectrum of traumatic, medical, surgical, and pediatric emergencies.

### Participants

In this study, the term “emergency physicians” refers to all doctors working in the ED, including both emergency medicine consultants and residents.

The target population included all emergency physicians working in the hospital’s ED during the data collection period, including consultants and resident physicians. Physicians practicing outside of emergency medicine were excluded.

At the time of the study, 25 physicians were working in the ED. A total population sampling approach was used. All 25 physicians working in the ED were invited to participate. Prior to the main survey, the questionnaire was piloted among three physicians, who were subsequently excluded from the final analysis, leaving 22 eligible participants. Participation was voluntary, and electronic informed consent was obtained before participants commenced the questionnaire.

### Survey instrument

Data were collected via a structured, self-administered questionnaire developed specifically for this study. The instrument was adapted from previously published and validated surveys assessing emergency physicians’ views on unnecessary management and diagnostic testing in international settings [22]. The questionnaire was modified to focus specifically on diagnostic imaging practices and contextualized to the Somali emergency care environment.

The final questionnaire consisted of 26 items organized into four domains:

1. **Demographic and professional characteristics**: age, gender, professional role, years of practice, shift type, compensation model, and workplace type. In this study, “years in emergency practice” referred to the total duration of clinical experience in emergency medicine following medical school graduation, including residency training.
2. **Perceptions of overuse**: frequency and seriousness of unnecessary imaging, perceived responsibility to avoid it, and common scenarios (e.g., CT head for minor trauma, CT pulmonary angiography for low-risk pulmonary embolism). Ultrasound underuse was also included to assess whether physicians perceived a shift toward increased CT use at the expense of appropriate first-line ultrasound in certain clinical scenarios.
3. **Drivers of overuse**: factors such as malpractice concerns, diagnostic uncertainty, patient or family requests, consultant pressure, time limitations, lack of system integration, and financial incentives.
4. **Optimization strategies**: perceived usefulness of patient education, shared decision-making, clinical decision rules, radiology consultation, audit and feedback, or malpractice reform.

Most attitudinal items used Likert-type response scales. Agreement-based questions were measured using a five-point scale (strongly agree, agree, neutral, disagree, strongly disagree), while frequency items used a five-point scale ranging from “always” to “never.” Drivers of imaging overuse were assessed using a three-point scale (major reason, minor reason, rarely).

In addition, the questionnaire included a single open-ended item:

**“What is the most important change your ED could implement to reduce unnecessary imaging?”**

The questionnaire was piloted among three emergency physicians (excluded from the final analysis) to ensure clarity, comprehensibility, and cultural appropriateness. Minor revisions were made in response to feedback from the pilot phase.

### Data collection procedures

Data collection was conducted over a one-month period between September 2025 and October 2025. The survey was administered electronically via Google Forms, and a secure link was shared through departmental communication channels. Physicians were invited through internal ED communication platforms and direct messages.

The introductory page provided information about the study objectives, voluntary participation, and assurances of confidentiality. No identifying information was collected. Responses were automatically stored in a password-protected database accessible only to the research team.

### Sample size and sampling

A total population sampling strategy was employed. Of the 25 physicians working in the ED, three were excluded after participating in the pilot phase, leaving 22 eligible physicians for the final survey.

In survey research, response rates of around 50% are generally regarded as acceptable, although higher rates are preferable to reduce the risk of nonresponse bias [23]. Accordingly, a minimum response rate of 50% was anticipated to ensure sufficient representation for descriptive analysis.

### Data analysis

All responses were exported from Google Forms into SPSS version 24 for statistical analysis. The analysis was descriptive, using frequencies and percentages to summarize participants’ demographic characteristics and survey responses.

Responses to the single open-ended question were reviewed and grouped into thematic categories based on recurring concepts, and the frequency of each theme was reported descriptively. This process was exploratory and intended to summarize common viewpoints rather than conduct a formal qualitative thematic analysis.

### Ethical considerations

This study was conducted in accordance with the ethical principles outlined in the Declaration of Helsinki. Ethical approval was obtained from the Institutional Review Board of Mogadishu, Somalia–Turkey Recep Tayyip Erdoğan Training and Research Hospital (Ethics Protocol No. MSTH/1284/2025).

Participation was voluntary, and electronic informed consent was obtained before participants commenced the questionnaire. Participant anonymity was maintained throughout the study. No personally identifiable information was collected or disclosed. The study posed no risks to participants, as it relied solely on self-reported perceptions.

## Results

### Sociodemographic characteristics of the participants

Of the 22 eligible emergency physicians, 21 completed the survey, yielding a response rate of 95.5%. The majority were aged 20–35 years (90.5%), and most were male (81.0%). Slightly more than half were consultants/specialists (52.4%), while the remainder were primarily residents. Regarding experience, most physicians had 3–5 years (42.9%) or 0–2 years (33.3%) of emergency practice. The vast majority (90.5%) worked mixed shifts and were employed at a tertiary teaching hospital (76.2%). Most participants were salaried (90.5%), with only a small proportion receiving fee-for-service compensation (Table 1).

**Table 1:** Sociodemographic characteristics of the participants (*n* = 21)

Variable	Category	Frequency (%)
Age (years)	20–35	19 (90.5%)
	35–50	2 (9.5%)
Sex	Male	17 (81.0%)
	Female	4 (19.0%)
Current role	Consultant/Specialist	11 (52.4%)
	Resident	9 (42.9%)
	General Practitioner	1 (4.8%)
Years in emergency practice	0–2	7 (33.3%)
	3–5	9 (42.9%)
	6–10	4 (19.0%)
	> 10	1 (4.8%)
Shift pattern	Mixed	19 (90.5%)
	Day only	1 (4.8%)
	Day + Night	1 (4.8%)
Primary workplace	Tertiary teaching hospital	16 (76.2%)
	Governmental hospital	3 (14.3%)
	Private hospital	2 (9.5%)
Compensation type	Salary	19 (90.5%)
	Fee-for-service	2 (9.5%)

### Patterns and drivers of diagnostic imaging overuse

A considerable proportion of physicians self-reported ordering unnecessary tests either daily (38.1%) or several times per week (28.6%), indicating that overuse was perceived as frequent. However, perceptions of the seriousness of unnecessary testing varied, and a majority of participants (57.1%) selected “prefer not to answer.” Regarding specific imaging scenarios, most physicians perceived CT head for minor adult injury (61.9%) and minor pediatric injury (76.2%) as overused. Opinions were more evenly divided regarding CT pulmonary angiography for low-risk PE, with responses distributed across agreement and disagreement categories. Most participants did not perceive ultrasound as underused (Table 2).

**Table 2:** Self-reported perceptions and frequency of diagnostic overuse (Likert-type scales) (*n* = 21)

Variable	Category	Frequency (%)
Unnecessary test ordering	Every day	8 (38.1%)
	Several times per week	6 (28.6%)
	Less than once per month	3 (14.3%)
	Unsure	4 (19.0%)
Unnecessary testing as a problem	Severe problem	4 (19.0%)
	Not too serious problem	4 (19.0%)
	Not a problem at all	1 (4.8%)
	Prefer not to answer	12 (57.1%)
CT head – minor adult injury	Agree	10 (47.6%)
	Strongly agree	3 (14.3%)
CT head – minor pediatric injury	Agree	14 (66.7%)
	Strongly agree	2 (9.5%)
CT pulmonary angiography (CTPA) for low-risk PE	Agree	7 (33.3%)
	Disagree	6 (28.6%)
	Strongly disagree	3 (14.3%)
	Strongly agree	1 (4.8%)
Ultrasound underuse	Disagree	8 (38.1%)
	Strongly disagree	3 (14.3%)
	Agree	4 (19.0%)
	Strongly agree	2 (9.5%)

The most frequently self-reported drivers of unnecessary imaging were fear of missed diagnosis (66.7%), requests from consultants or admitting teams (57.1%), and departmental culture (52.4%). Malpractice concerns and patient expectations were also commonly identified factors (each 42.9%). Financial incentives were the least influential, with only **9.5%** considering them a major reason (Table 3).

**Table 3:** Self-reported reasons contributing to diagnostic overuse (3-point Likert-type scale) (*n* = 21)

Factor	Major reason (%)	Minor reason (%)	Rarely (%)
Fear of missed diagnosis	14 (66.7%)	3 (14.3%)	3 (14.3%)
Malpractice concerns	9 (42.9%)	5 (23.8%)	4 (19.0%)
Patient/family expectations	9 (42.9%)	11 (52.4%)	–
Consultant/admitting team requests	12 (57.1%)	7 (33.3%)	–
Limited consultation time	9 (42.9%)	5 (23.8%)	3 (14.3%)
Night-shift/limited imaging access	6 (28.6%)	5 (23.8%)	10 (47.6%)
Departmental culture	11 (52.4%)	3 (14.3%)	5 (23.8%)
Patient reassurance	7 (33.3%)	6 (28.6%)	8 (38.1%)
Imaging improves ED flow	7 (33.3%)	10 (47.6%)	3 (14.3%)
Financial/reimbursement incentive	2 (9.5%)	6 (28.6%)	9 (42.9%)

### Strategies and decision-support use

Most participants considered patient education, shared decision-making, and more comprehensive clinical assessment to be helpful strategies for reducing unnecessary imaging. Physician education and malpractice reform were the most strongly endorsed interventions, with 71.4% rating them as very or extremely helpful. Radiology consultation, image-sharing systems, and feedback on ordering behavior were also viewed favorably by a substantial proportion of participants (Table 4).

**Table 4:** Perceived usefulness of strategies to reduce unnecessary imaging (5-point Likert-type scale) (*n* = 21)

Strategy	Very/Extremely helpful (%)	Moderately (%)	Slightly/Not (%)
Patient/family education + shared decision-making	10 (47.6%)	5 (23.8%)	6 (28.6%)
Complete systemic assessment + more consultation time	13 (61.9%)	3 (14.3%)	5 (23.8%)
Physician education + malpractice reform	15 (71.4%)	1 (4.8%)	5 (23.8%)
Easy radiology consultation + image-sharing	12 (57.1%)	3 (14.3%)	6 (28.6%)
Feedback on ordering + remove incentives	10 (47.6%)	7 (33.3%)	4 (19.0%)

The self-reported use of clinical decision rules varied across scenarios. For adult head injuries, most participants reported using decision rules at least sometimes, with 71.4% indicating use sometimes or often. Similar patterns were observed for pediatric head injuries and suspected pulmonary embolism. Approximately 47.6% of participants reported having imaging decision-support tools integrated into their electronic health record systems (Table 5).

**Table 5:** Use of clinical decision rules and EHR decision support (*n* = 21)

Rule/System	Always (%)	Often (%)	Sometimes (%)	Rarely (%)	Never (%)
CCHR/New Orleans (adult head)	1 (4.8%)	7 (33.3%)	8 (38.1%)	3 (14.3%)	2 (9.5%)
PECARN (pediatric head)	2 (9.5%)	5 (23.8%)	7 (33.3%)	3 (14.3%)	4 (19.0%)
Wells/PERC + D-dimer (PE)	7 (33.3%)	4 (19.0%)	6 (28.6%)	1 (4.8%)	3 (14.3%)
EHR imaging decision support	Yes 10 (47.6%)	No 11 (52.4%)	–	–	–

CCHR: Canadian CT Head Rule; PECARN: Pediatric Emergency Care Applied Research Network; PERC: Pulmonary Embolism Rule-out Criteria

In the open-ended responses, physicians highlighted several key strategies to reduce unnecessary imaging. The most common themes were the need for more comprehensive clinical assessment before imaging (28.6%), physician education and continuous training (19.0%), and the integration of decision-support tools (14.3%). Other suggestions included improved interdepartmental collaboration, malpractice reform, increased use of ultrasound, and workflow improvements (Table 6). A minority of participants (2, 9.5%) advocated policy reforms and interdepartmental collaboration to enhance accountability and multidisciplinary communication. In comparison, another 2 (9.5%) identified malpractice reform and stronger medico-legal frameworks as essential for mitigating defensive testing. Isolated responses included increased use of ultrasonography (4.8%), reduced financial incentives (4.8%), and workflow modifications to enhance the management of busy shifts (4.8%) (Table 6).

**Table 6:** Thematic summary of physicians’ suggested changes to reduce unnecessary imaging (*n* = 21)

Thematic Category	Example Responses	Frequency (%)
Comprehensive clinical assessment before imaging	“Full history and examination before testing”; “Base imaging on differential diagnosis”	6 (28.6%)
Physician education and continuous training	“Education, training, and accountability”; “Improve doctors’ knowledge.”	4 (19.0%)
Decision support tools and evidence-based guidelines in EHR	“Integrate CDS tools and evidence-based rules in EMR workflow.”	3 (14.3%)
Policy and interdepartmental collaboration	“Improve collaboration between specialties”; “Departmental policies to reduce overuse.”	2 (9.5%)
Malpractice reform/medico-legal clarity	“Develop malpractice guides”; “Improve legal protection for physicians.”	2 (9.5%)
Promote ultrasound as first-line imaging	“We have to use more ultrasound.”	1 (4.8%)
Reduce financial incentives/align rewards	“Train physicians to decrease incentives.”	1 (4.8%)
Improve ED workflow and staffing	“Address imaging during busy shifts.”	1 (4.8%)
Other/not specified	“Nothing,” “More ideas”	1 (4.8%)

## Discussion

This work represents one of the first efforts to examine and contextualize emergency physicians’ perspectives on the overuse of advanced diagnostic imaging in Somalia. The study assessed the relevance of previously identified personal and institutional drivers of unnecessary testing and incorporated exploratory insights from open-ended physician responses. The findings indicate that many physicians recognize overuse as a common practice, primarily driven by concerns about missed diagnoses, diagnostic uncertainty, and pressure from consultants. These findings are consistent with global studies conducted in high- and middle-income settings. Moreover, the study highlights the contextual challenges faced within Somalia’s resource-limited healthcare system.

### Perceptions and frequency of diagnostic overuse

This study highlights that most emergency physicians recognize diagnostic imaging overuse as a persistent issue in their daily practice, with many reporting that they regularly order unnecessary imaging. Similar patterns have been observed internationally, in which physicians report frequent use of low-value imaging despite awareness of its limited clinical benefit [24]. These findings underscore that diagnostic overuse remains a significant global challenge, even among physicians who are aware of its risks.

Recent post-pandemic studies have reported notable shifts in imaging practices in EDs. For example, one study observed increased chest computed tomography (CT) utilization and a younger population being imaged after the COVID-19 pandemic, indicating changes in diagnostic behavior and patient demographics [25]. Another analysis reported a sustained post-pandemic surge in chest and multi-region CT examinations in the ED, even after pandemic-related restrictions were lifted [26]. In addition, radiology reports have been shown to serve as real-time epidemiological data, with chest CT findings correlating with regional COVID-19 case trends, highlighting the expanded role of imaging beyond individual diagnosis during infectious outbreaks [27]. Together, these findings suggest that evolving clinical uncertainty, changes in patient profiles, and the epidemiological role of imaging may contribute to persistent increases in CT utilization in emergency settings.

The reluctance of more than half of the participants to classify overuse as a serious healthcare system problem may reflect professional discomfort or institutional barriers to acknowledging inefficiencies within the system. Similar attitudes have been documented among physicians in low-resource environments, where hierarchical culture and limited feedback mechanisms restrict discussions on quality improvement [28]. Evidence from middle- and high-income healthcare settings also indicates that physicians may hesitate to acknowledge imaging overuse due to defensive medicine practices, institutional culture, and concerns about professional evaluation [9, 29]. The recognition that CT head scans for minor adult and pediatric trauma are commonly overused is consistent with global evidence showing that head CT is among the most frequently ordered unnecessary imaging tests [30]. Despite the availability of guidelines, emergency clinicians often overuse CT scans to mitigate diagnostic uncertainty or to reassure themselves and patients [8]. This pattern suggests the need for structured institutional guidelines and consistent audit systems to guide imaging use in Somalia’s EDs.

### Reasons contributing to diagnostic overuse

Emergency physicians often request imaging because of concerns about missed diagnoses, malpractice risk, and patient expectations. In addition, diagnostic uncertainty, time pressure, and institutional cultures that equate more imaging with safer care further contribute to this behavior [10, 29, 31].

The dominant factors identified, including fear of missed diagnosis, requests from consultants or the admitting team, and departmental culture, are well-recognized drivers of overuse in emergency medicine [9]. Studies have shown that emergency physicians often practice defensively, particularly in environments with limited supervision, peer review, or clear clinical pathways [32].

In the study setting, residents are permitted to order diagnostic imaging independently as part of routine clinical practice, although consultants provide overall supervision. This structure may contribute to variability in imaging utilization, particularly in busy emergency settings. In Somalia, diagnostic uncertainty is compounded by shortages of specialists and inconsistent access to radiology consultations, promoting reliance on advanced imaging even for low-risk cases.

Interestingly, financial incentives were reported as the least influential factor. This contrasts with evidence from high-income countries, where reimbursement models and profit-driven systems are significant contributors to imaging overuse [33]. The absence of such motivation in the Somali public hospital context reinforces the notion that systemic and cognitive drivers, rather than economic incentives, play a greater role.

Additionally, patient or family pressure was cited by nearly half of the participants, consistent with findings from international surveys showing that patient expectations often compel clinicians to perform imaging “for reassurance” rather than for medical necessity [34]. Addressing such expectations through communication and education may therefore be a key component of reducing unnecessary testing.

### Strategies to reduce diagnostic overuse

The participants strongly endorsed patient education, shared decision-making, malpractice reform, and physician training as effective strategies to reduce overuse. These findings align with evidence that patient-centered discussions and informed consent lessen the demand for unnecessary imaging and enhance trust in clinical judgment [35].

The high value placed on education and extended consultation time reflects the understanding that overuse often arises from rushed decision-making in overcrowded emergency settings. Similar studies have demonstrated that incorporating structured clinical decision tools and feedback programs into routine workflows reduces low-value imaging without increasing the number of missed diagnoses [36].

Furthermore, physicians viewed radiology consultation and peer feedback as beneficial interventions. Peer comparison is a powerful behavioral nudge in changing ordering patterns [37]. When clinicians receive feedback comparing their imaging rates with those of peers, ordering rates typically decrease over time. Establishing such audit-feedback systems could therefore be a feasible and low-cost strategy in Somalia’s tertiary hospitals.

To summarize these proposed interventions more clearly, the key strategies identified in the literature are presented in Table 7.

**Table 7:** Summary of strategies to reduce unnecessary diagnostic imaging in emergency departments

Strategy	Rationale	Evidence from literature
Patient education and shared decision-making	Helps manage patient expectations and reduce demand for unnecessary imaging	Improves patient understanding and reduces low-value testing
Physician education	Improves awareness of imaging guidelines	Training programs reduce unnecessary imaging
Clinical decision rules	Standardizes imaging decisions	Tools such as CCHR and PECARN reduce CT use
Radiology consultation	Supports appropriate imaging selection	Collaboration improves diagnostic stewardship
Audit and feedback	Provides physicians with performance comparisons	Peer comparison reduces imaging ordering
Comprehensive clinical assessment	Encourages reliance on clinical reasoning before imaging	Structured assessment reduces unnecessary testing

### Use of evidence-based clinical decision rules

Although many emergency clinicians are aware of validated decision rules, multiple studies have shown that their use is inconsistent, often owing to implementation barriers such as a lack of reminders, poor workflow integration, limited health information infrastructure, and the absence of institutional mandates [38–40]. Approximately half of the participants reported that decision support was integrated into their EHRs, suggesting incomplete adoption of digital tools. However, previous studies have shown that embedding clinical decision support into EHR workflows can reduce utilization of high-cost imaging and improve adherence to evidence-based guidelines [41]. Evidence shows that embedding these decision rules directly into EHR systems and providing automated reminders can significantly increase adherence and reduce unnecessary CT use [42]. The relatively frequent use of Wells/PERC compared with head injury rules may indicate greater confidence in adult medical algorithms than trauma protocols do. Targeted training emphasizing pediatric and trauma-specific guidelines could therefore enhance the rational use of imaging in these domains.

### Suggested changes to reduce unnecessary imaging

The open-ended responses reflected physicians’ awareness of practical interventions that could be implemented locally to address overuse. The most common suggestion to strengthen clinical assessment before test ordering highlights physicians’ acknowledgment that reliance on imaging has eroded clinical reasoning. Similar findings have been reported in studies showing that structured examinations and differential diagnoses reduce unnecessary imaging orders [43, 44].

The participants’ emphasis on education, the integration of decision support tools, and policy reform underscores the need for system-level change rather than solely individual responsibility. The implementation of local imaging appropriateness guidelines, supported by continuing education and EHR integration, has demonstrated success in improving diagnostic stewardship in other healthcare systems through tools such as the Canadian CT Head Rule (CCHR) [45].

A smaller group advocated for workflow reorganization and malpractice reform, aligning with global recommendations for institutional accountability frameworks and supportive governance models [46–49]. These measures, coupled with enhanced interdepartmental collaboration between emergency medicine and radiology, could yield sustainable improvements in imaging utilization.

### Strengths and limitations

This study is among the first to examine emergency physicians’ perspectives on diagnostic imaging overuse in Somalia, providing context-specific insights from a tertiary-referral ED. The use of a total-population sampling approach and a high response rate enhanced the representativeness of the findings in the study setting. Additionally, the inclusion of an open-ended question allowed participants to provide exploratory, context-specific suggestions beyond predefined survey options.

However, several limitations should be considered. First, the study was conducted at a single tertiary hospital, which may limit the generalizability of the findings to other healthcare settings within Somalia or similar contexts. Second, the sample size was relatively small (*n* = 21); however, this reflects the limited number of emergency physicians in the study setting rather than a sampling constraint, as a total population approach was used and nearly all eligible physicians participated. Third, the results were based on self-reported perceptions and practices, which may be subject to recall and social desirability bias, particularly for sensitive items such as whether imaging overuse is considered a serious problem. In addition, although the response rate was high, the possibility of non-response bias cannot be completely excluded. Finally, although the survey included an open-ended question, these qualitative responses were grouped into themes for descriptive reporting rather than analyzed using a formal qualitative approach. These limitations suggest that the findings should be interpreted as exploratory and context-specific, and caution should be exercised when generalizing the results beyond similar settings.

### Clinical and research implications

The findings highlight the need for targeted interventions to reduce unnecessary imaging in ED, particularly those addressing diagnostic uncertainty, consultant-driven requests, and defensive practices. Strategies such as physician education, implementation of clinical decision rules, improved radiology consultation, and feedback on ordering behavior may help optimize imaging use in similar resource-limited settings.

Future research should involve larger, multicenter studies to validate these findings across different healthcare contexts. Studies incorporating objective imaging utilization data and formal qualitative methods could further clarify the drivers of overuse and inform tailored interventions.

## Conclusion

This study demonstrates that emergency physicians in this setting are aware of diagnostic imaging overuse, recognize its major contributing factors, and are receptive to evidence-based and system-level strategies to improve practice. However, the absence of national imaging policies, clinical decision support systems, and continuous audit mechanisms remains a critical barrier to progress.

## Data Availability

The datasets generated and/or analyzed during the current study are available from the corresponding author on reasonable request.

## Abbreviations

CT: Computed Tomography
CTPA: Computed Tomography Pulmonary Angiography
EHR: Electronic Health Record
ED: Emergency Department
LMICs: Low–and Middle–Income Countries
PECARN: Pediatric Emergency Care Applied Research Network
CCHR: Canadian CT Head Rule
PE: Pulmonary Embolism
SPSS: Statistical Package for the Social Sciences
MRI: Magnetic Resonance Imaging
WHO: World Health Organization
CDS: Clinical Decision Support
EMR: Electronic Medical Record
PERC: Pulmonary Embolism Rule–out Criteria
COVID: 19–Coronavirus Disease 2019
